# Next generation plant biostimulants & genome sequencing strategies for sustainable agriculture development

**DOI:** 10.3389/fmicb.2024.1439561

**Published:** 2024-07-17

**Authors:** Shivanshu Garg, Pooja Nain, Ashish Kumar, Samiksha Joshi, Himanshu Punetha, Pradeep Kumar Sharma, Sazada Siddiqui, Mohammed O. Alshaharni, Uthman Balgith Algopishi, Amit Mittal

**Affiliations:** ^1^Department of Biochemistry, CBSH-GBPUA&T, Pantnagar, India; ^2^Department of Soil Science, College of Agriculture, GBPUA&T, Pantnagar, India; ^3^Department of Microbiology, CBSH-GBPUA&T, Pantnagar, India; ^4^School of Agriculture, Graphic Era Hill University, Bhimtal, India; ^5^Department of Environment Science, Graphic Era Deemed to be University, Dehradun, India; ^6^Department of Biology, College of Science, King Khalid University, Abha, Saudi Arabia; ^7^School of Allied Sciences, Graphic Era Hill University, Bhimtal, India

**Keywords:** plant biostimulants, biomolecules, next-generation sequencing, metabolites, sustainable

## Abstract

The best environment for plant growth and development contains certain essential metabolites. A broad category of metabolites known as “plant biostimulants” (PBs) includes biomolecules such as proteins, carbohydrates, lipids, and other secondary metabolites related to groups of terpenes, specific nitrogen-containing compounds, and benzene ring-conjugated compounds. The formation of biomolecules depends on both biotic and abiotic factors, such as the release of PB by plants, animals, and microorganisms, or it can result from the control of temperature, humidity, and pressure in the atmosphere, in the case of humic substances (HSs). Understanding the genomic outputs of the concerned organism (may be plants or others than them) becomes crucial for identifying the underlying behaviors that lead to the synthesis of these complex compounds. For the purposes of achieving the objectives of sustainable agriculture, detailed research on PBs is essential because they aid in increasing yield and other growth patterns of agro-economic crops. The regulation of homeostasis in the plant-soil-microbe system for the survival of humans and other animals is mediated by the action of plant biostimulants, as considered essential for the growth of plants. The genomic size and gene operons for functional and regulation control have so far been revealed through technological implementations, but important gene annotations are still lacking, causing a delay in revealing the information. Next-generation sequencing techniques, such as nanopore, nanoball, and Illumina, are essential in troubleshooting the information gaps. These technical advancements have greatly expanded the candidate gene openings. The secondary metabolites being important precursors need to be studied in a much wider scale for accurate calculations of biochemical reactions, taking place inside and outside the synthesized living cell. The present review highlights the sequencing techniques to provide a foundation of opportunity generation for agricultural sustainability.

## 1 Introduction

The plant-based biochemicals, especially secondary metabolites, when released in a local environment influence the growth of other species of plants by combating pathogens and increasing the chances of survival of plants. These compounds providing adequate growth to plant species are termed as plant biostimulants. Agriculture is a complicated discipline concerning the types of biochemicals involved because of the interaction between natural processes. Although the growth of agriculture depends on natural processes such as weather, soil conditions, and fertilizers, human-led effort generates efficient and higher productivity while utilizing minimum agents, which is harmful for the mother nature. This motivates the idea of “sustainable agriculture” as a potential force for human existence and the sustainability of other ecological niches (Divekar et al., 2022).

However, nature does not always support the agriculture sector in a beneficial manner. Pathogens have a wide range of families, specific orders, and overlapping population of species, such as larvae, caterpillars, and moths, which feed on plants only in the form of sap or leaves. The natural selection is followed by organisms according to modification in the set of genes present in their genome that actually develops the ultimate phenotypic effect. However, continuous feeding on plants without any defense mechanism developed by plants will destroy the plant species. This may be further accompanied by pathogenic species of fungi, bacteria, and viruses. If this continues for long, the plants will be extinct, but this is not occurred irrespective of the presence of pathogen and plant feeders because of the biochemicals released by plants in the micro-environment leading to their substantial growth. This leads to development of the concept of plant biostimulants. However, other chemical compounds which deter pathogen’s growth and favor plant’s growth may also be termed as plant biostimulants. However, chemical interaction is the finest strategy used by living organisms to maintain an overall equilibrium among them. When it comes to the growth of plants, this becomes a plant biostimulant for insects, an insect biostimulant, and fungus, a fungal biostimulant. However, the terms “insect growth retardant” and “fungal growth retardant,” as well as the agents, added to the accurate definition of “plant biostimulant” because they are a part of removing the potential risks to the affected plant. The importance of sustainable agriculture is now brought up in relation to plant biostimulants, whose next generation potential can be used for the opportunities of sustainable agriculture (Nephali et al., 2020).

### 1.1 Agriculture and plant biostimulants

The agricultural sector has recently been confronted with the simultaneous challenges of maximizing resource utilization, minimizing environmental impact on the ecosystem, and an expanding global population. Chemical fertilizers and pesticides are crucial for agriculture, as they provide cultivators a strong method for yield and consistent efficiency throughout the seasons in both ideal and sub-standard circumstances. To boost the long-term viability of agricultural production systems, a number of technological advancements have been proposed over the past three decades. These advancements have significantly reduced the use of synthetic agrochemicals such as pesticides and conventional fertilizers. The utilization of regular plant biostimulants (PBs) that have beneficial impacts on the agricultural sector increased the crop efficiency and nutrient use efficiency (NUE), with higher potential of flower blooming, leading to productive fruit development or pod set, plant root development, enhancement in bio-organic product set, and imparting resistance to an extensive variety of biotic and abiotic stressors leading to adequate optimum set of growth conditions for the ecosystem advancement (Colla and Rouphael, 2015). Fertilizers and plant protection products were initially excluded from the definition of PBs. Researchers from Virginia Polytechnic Institute and State University’s Department of Crop and Soil Environmental Sciences defined plant biostimulants (PBs) as compounds that stimulate plant growth when applied in small amounts (Zhang and Schmidt, 1997). While fertilizers and soil amendments also promote plant development, they are usually administered in higher quantities. The scientists used the phrase “minute quantities” to distinguish biostimulants from other substances. It was hypothesized that the PBs discussed in this article primarily affect plants. In 2012, the European Commission assigned an *ad hoc* study on PBs to evaluate the materials and substances involved. du Jardin (2015) published this study as: “A Bibliographic Investigation of the Study on Plant Biostimulants.” The accompanying definition was proposed in light of the logical writing (250 logical articles that utilized the expression “biostimulant” in their titles and abstracts). According to the definition, “plant biostimulants refer to substances and materials that, when administered to plants, seeds, or growing media in specific formulations, can alter physiological processes in plants, potentially enhancing growth, development, and/or responses to stress.” The only exceptions to this definition are pesticides and nutrients. According to the research by du Jardin (2015), PB is made up of a wide range of materials, and he identified eight categories of substances that serve as biostimulants. Chemical elements (Al, Co, Na, Se, and Si), inorganic salts such as phosphate and seaweed extracts (brown, red, and green macroalgae), chitin and derivatives (Ghasemi Pirbalouti et al., 2017), anti-transpirants such as kaolin and polyacrylamide, free amino acids and substances that contain nitrogen (peptides, polyamines, and betaines), humic materials, complex organic materials (from sewage) did not have any biostimulants for microorganisms. After 3 years, in the context of “*Biostimulants in Horticulture*” by Colla and Rouphael (2015) and du Jardin (2015) proposed a new definition. Scientific evidence regarding the mode of action, nature, and types of effects that PBs have on agricultural and horticultural crops supported this definition. As indicated by the definition of PBs by du Jardin (2015), “any substance or microorganism that is applied to plants fully intent on further developing sustenance proficiency, abiotic stress resilience, is a plant biostimulant, no matter what its supplement content.” This definition could be completed by defining PBs as “*commercial products containing mixtures of such substances and/or microorganisms*.” The definition of PBs has been the subject of intense debate for the past 10 years. Most recently, the following was established by the brand-new Regulation (EU) 2019/1009: An EU compost that revives plant nourishment processes regardless of the supplement content is considered to be plant biostimulant. The purpose is to enhance one or more of the following plant or plant rhizosphere characteristics: (i) the efficiency with which nutrients are utilized, (ii) tolerance to abiotic stress, (iii) the quality characteristics, and (iv) bioavailability of restricted nutrients in the soil or rhizosphere (EU, 2019). To account for broad range, six non-microbial and three microbial PB categories were proposed: (i) humic and fulvic acids (Canellas et al., 2015) (ii) chitosan (Pichyangkura and Chadchawan, 2015) (iii) protein hydrolysates (Gómez-Merino and Cand-Trejo-Téllez, 2015) (iv) phosphites (Varadarajan et al., 2002) (v) seaweed extracts (Battacharyya et al., 2015) (vi) silicon, and (vii) arbuscular mycorrhizal fungi (AMF) (Rouphael et al., 2015), (viii) plant growth-promoting rhizobacteria (PGPR) (Ruzzi and Aroca, 2015; Omidbakhshfard et al., 2019), and (ix) *Trichoderma* spp. (López-Bucio et al., 2015).

Along with all these aspects covered by plant biostimulants, the growth of the agriculture sector clearly relies on biochemical interactions among different species of plants. These interactions proved useful in maintaining the healthy soil conditions, elongated root zone growth, and less pathogenic condition development while minimizing the use of harmful chemical fertilizers, simultaneously achieving the goal of sustainable agriculture.

### 1.2 The collective data potential

To understand any concept, data generation should be influenced by a set of reasons to determine the concerned research so far across the world. In the case of newly developing field biostimulants, more than 700 scientific studies were published between 2009 and 2019. These studies demonstrate that non-microbial and morpho-anatomical, biochemical, physiological, and molecular plant responses, such as an increase in crop productivity and tolerance to abiotic stresses, can both be induced by PBs (Calvo et al., 2014; Haplern et al., 2015; Nardi et al., 2016; Yakhin et al., 2017; Rouphael et al., 2017a,b,c, 2018a,b; De Pascale et al., 2018).

**Figure 1:**
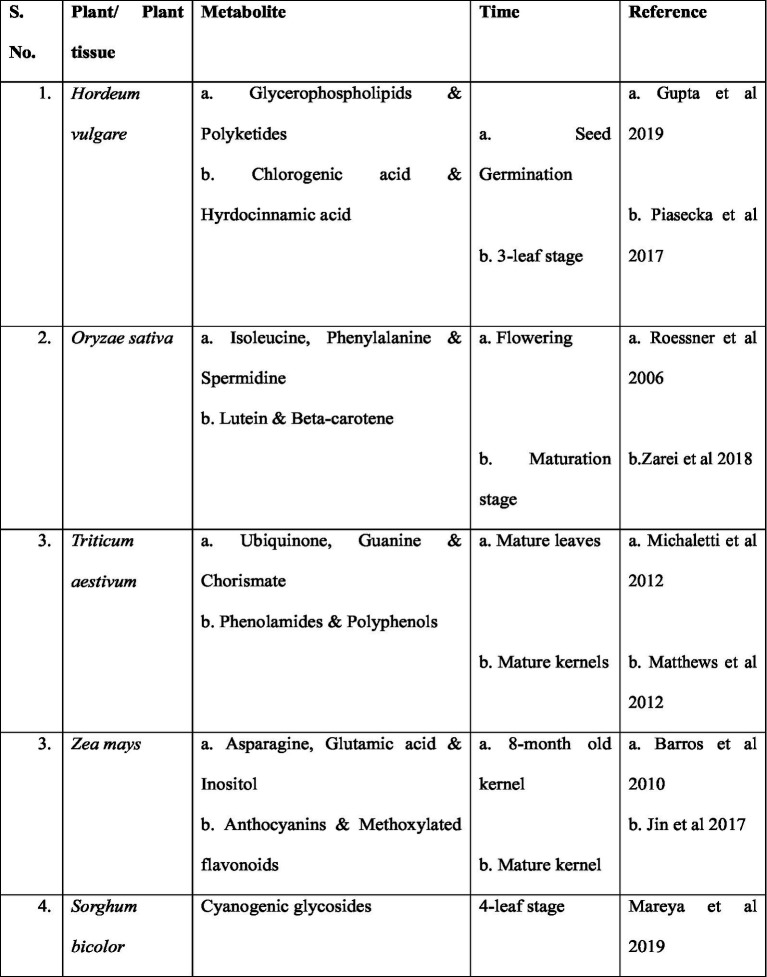
Cycle of plant biostimulants in nature. This figure represents how the components of nature are regulated by plant biostimulants and the links are established between them.

Information presented here denotes the collective data on 50 scientific studies from highly qualified research studies removing PBs and covering sub-atomic, cellular, and physiological components of fundamental plant biostimulant associations under variable agro-climate zones. In addition, several topics that will be helpful to the scientific community, extension specialists, and business organizations in deciphering the causal/functional mechanism of both microbial and non-microbial biostimulants are discussed. Currently, there is a market trend toward formulations containing both microbial and non-microbial plant biostimulants. However, many of these commercial products lack sufficient scientific evidence to validate their synergistic effects. Therefore, it is imperative to use robust experimental approaches to scientifically characterize the properties of these formulations and their individual impact on crop yield and resistance (Rouphael and Colla, 2020). This future challenge lies in the research, development, and innovation of second-generation PB. The advantages of PB (Figure 1) reveal the potentials of maintaining the macro-ecosystem (concerned with human influence) and micro-ecosystem (concerned with rhizosphere). The second era of biostimulants in which cooperative energies and corresponding systems can be practically planned should be possible by explaining the rural capability (i.e., to further develop the supplements that used productivity, quality, and resilience to the abiotic stresses) and activity components of PBs.

### 1.3 Challenges for sustainable agriculture

The major challenges to be encountered for in this direction are biodiversity conservation, protecting the environment from degradation. Wherein habitat expansion that is needed for crops meant to be grown for food supply especially rice, wheat, and maize (de Azevedo et al., 2019), rehabilitation must be ensured (for crops that were grown but not in an adequate amount). Legislation for genetic resources is required, wherein the planned use of genetic materials is carried out through breeding innovations whether classical or modern. On one hand, clearing the forest lands for agricultural production opens the gateways of feeding the over growing population and, on the other hand, overburdens the nature by reducing photosynthetic crops, depleting natural niches and habitats of beneficial animal species, and drastically reducing the natural mineral content of soil. To cope this adequate measures to be taken monitored by government and private organizations and the training and conference programs at national and international levels and self-consciousness among people to protect the forest land. Another major difficulty to forest land is industrialization which depletes cultivated soil and increases pollutants in air and water, thus hampering the agriculture sector in a more drastic way. However, natural calamity such as flood is responsible for soil erosion, but they are not frequent until and unless the forest land is under devastation by human-generated needs. The extension education sector should clearly define the impacts in general body meetings, where the concern for possibility of such mishaps should be clearly described to people ultimately raising the environment-friendly issues.

## 2 Maneuvering classification data on biostimulants

In view of where the biostimulants are derived from, these biostimulants are divided into two distinct categories. All products with a biological origin, such as those derived from microorganisms or the plant itself, are under one category (biotic biostimulants), and all products without a biological origin, such as those derived from chemicals and physical factors, are under the second category (abiotic biostimulants). In addition, biotic biostimulants may include molecules with a known structure and have a particular composition, or they may be more complex and include multiple molecules with distinct structures. Additionally, biostimulant products can be categorized into two types: microbial, derived from beneficial microbes such as arbuscular mycorrhizal fungi (Giovannini et al., 2020) and plant growth-promoting bacteria, and non-microbial, derived from the extracts of plant microalgae, humic substances, and biopolymers, such as chitosan. This is another way to classify biostimulant products (Lugtenberg and Kamilova, 2009; Ahemad and Kibret, 2014; Pérez-Montaño et al., 2014; de Vries et al., 2020; Rouphael and Colla, 2020). In particular, there are several ways in which the microbial biostimulants may promote plant growth (Kumari et al., 2022). Biofertilization, root growth stimulation, tolerance to plant stressors, and rhizoremediation are examples of direct effects on plant growth promotion as they involve the interaction between plant roots and biochemicals. On the other hand, plant pathogen control and increased enzymatic activity may indirectly stimulate plant growth (de Vries et al., 2020). Finally, a number of researchers make a distinction between non-phytohormonal and phytohormonal non-microbial biostimulants, which include compounds containing proteins (Geelen et al., 2020).

### 2.1 Crucial counterparts of PBs

Certain components that exist naturally within the plant have the potential of acting as a biostimulant, as shown in Table 1. The following section briefly discusses the structure of these crucial counterparts. Moreover, prominent examples are also found outside the plant, where another biological agent such as fungi is involved.

**Table 1:** Primary classification of plant biostimulants.

S. no.	Plant biostimulants	Key points	References
1.	N-containing compounds (amino acids) and protein hydrolysates (PHs)	A combination of peptides and amino acids is formulated using animal or plant proteins subjected to chemical, enzymatic, or thermal hydrolysis Enhance the physiological and biochemical processes of both primary and secondary plant metabolism Are able to mitigate abiotic stress’s negative effects	Ahmad et al. (2015) and Colla et al. (2015) Vioque et al. (2000) and Colla et al. (2014) Yakhin et al. Lubyanov (2017)
2.	Humic materials	Incorporate humic and fulvic acids, which have distinct characteristics of their own like sub-atomic weight, carbon content, and polymerization degrees They might increase the soil’s cationic exchange capacity (CEC) by interacting with root membrane transporters (Figure 3)	Nardi et al. (2016) Canellas et al. (2015)
3.	Extracted seaweeds	Brown seaweed extracts, including those from the *Ascophyllum*, *Fucus*, and *Laminaria* genera They have a lot of hormone-active compounds, polysaccharides, and polyphenols that help plants grow and develop	Khan et al. (2009) Craigie (2011) and González et al. (2012)
4.	Biopolymers (chitosans and other polymers)	Nematodes, fungi, insects, and crustaceans all naturally contain chitosans that improve plant root growth Control plant defense mechanisms that make plants more resistant to biotic and abiotic stressors by controlling the biosynthesis of phytoalexins, degradation and generation of reactive oxygen species (ROS) and pathogenic native proteins	Pichyangkura and Chadchawan, 2015 Ghasemi Pirbalouti et al. (2017)
5.	*Trichoderma*, rhizobium, and plant growth-promoting rhizobacteria (PGPR), which are both mycorrhizal and non-mycorrhizal fungi, are biostimulants for microorganisms	Symbolic fungi, particularly *Glomus*-genus arbuscular mycorrhizal fungi (AMF) induces nutrient channeling Genus *Trichoderma* have hyphae that stimulate plant iron uptake Beneficial bacteria, also known as PGPBs, that aid in plant growth (*Bacillus*, *Rhizobium* and *Pseudomonas*)	Pereira et al. (2019) and Petropoulos et al. (2019) López-Bucio et al. (2015) Ruzzi and Aroca, 2015
6.	Phosphite (Phi)	Phosphate analog (H_2_PO_4_) are weal acid compounds that affects a variety of plant development and growth processes by regulating water uptake Various vegetable crops chelate heavy metal ions and create nutrient channels within plant Biostimulatory effects on citrus, avocado, banana, peach, raspberry and strawberry fruits	Varadarajan et al. (2002) Rickard (2000), Lobato et al. (2011), Olivieri et al. (2012), Tambascio et al. (2014), and Oyarburo et al. (2015) Rickard (2000), Moor et al. (2009), and Estrada-Ortiz et al. (2013)
7.	Silicon	Resistant to both environmental stressors (biotic and abiotic) thus eventually participates in plant cell wall formation and provides rigidity to plant. Thus in all maintains overall physiology of plant structure, from roots to shoots	Colla and Rouphael (2015)
8.	Vermicomposts	Vermicompost leachates hormone activity as a result of their high concentration of hormone-like trace elements like cytokinins, indole-acetic acid, eighteen gibrellic acids and brassinosteroids Vermicompost contains phytohormones belonging to three distinct classes, including auxins gibberellins and cytokinins	Aremu et al. (2014) Zhang et al. (2015)

Due to their promising properties, protein hydrolysates, one of the various compounds with biostimulatory activity, are the focus of scientific investigation. In fact, these compounds are made up of soluble peptides and amino acids that are mostly made by heating or enzymatically making proteins from animals or plants (Carillo et al., 2019). The positive impacts of these products are attributed to the stimulation of metabolites associated with plant growth processes and the induction of hormone-like activities, both of which influence plant growth and productivity (Ertani et al., 2009; Colla et al., 2017). These hormones are derived from indole ring such as auxins, which help in encountering salt stress by plants while cytokinins helping the plant during water stress, abscisic acid encountering chilling stress, gibberellic acid helping in ion partitioning, and salicylic acid helping in biomass accumulation (Egamberdieva et al., 2017).

Over the past few decades, seaweed extracts, which are another well-known classes of biostimulants, have gradually become increasingly utilized in farming (Craigie, 2011). These compounds have been used across various crops due to their capability to enhance crop performance, promote tolerance to abiotic stressors, and prolong the shelf life of numerous crop products (Battacharyya et al., 2015). They are generally made up of earthy-colored ocean growth-forming brown algae such as *Ascophyllum nodosum*, *Ecklonia maxima*, and *Macrocystis pyrifera* (do Rosário Rosa et al., 2021), and they contain advancing chemicals or minor components such as iron (Fe), copper (Cu), zinc (Zn), and manganese (Mn) (Sivasankari et al., 2006; Gupta et al., 2011). Other dynamic biomolecules, such as phloroglucinol and eckol, come from the earthy-colored ocean growth-forming brown algae *Ecklonia maxima*, one of the most well-known Kelp species and is utilized as fluid manure (with reduced viscous properties) (Rengasamy et al., 2016). Additionally, the production of carrageenan from *Kappaphycus alvarezii* or other extracts from industrially processed seaweeds (such as carrageenan) may both reduce the carbon footprint of the industrial sector and increase the value of seaweeds (Ghosh et al., 2015).

**Figure 2:**
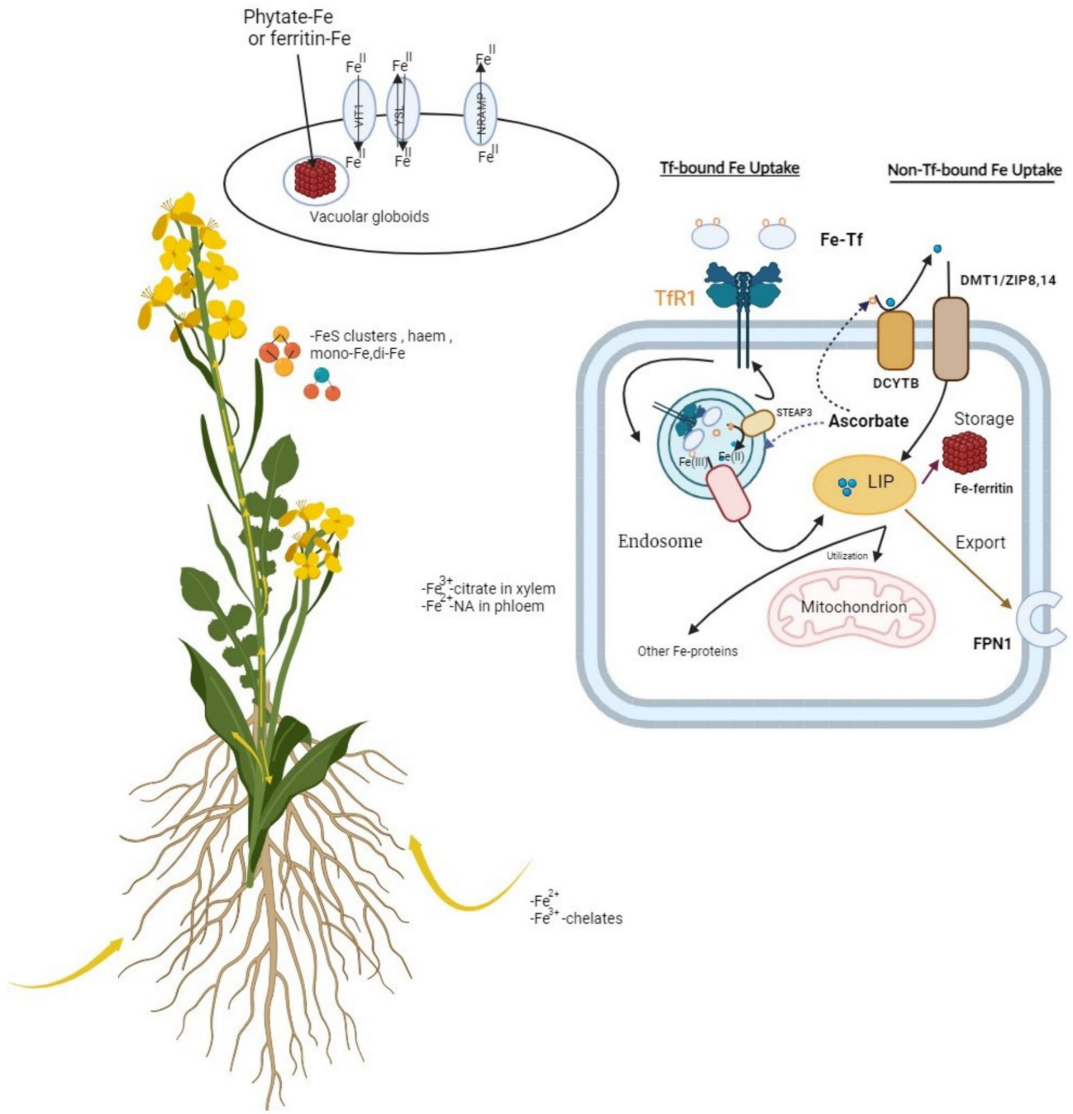
Involvement of ferritin genes in iron uptake: TfR (transport ferritin) membrane-bound transporters regulates the action of iron uptake in plants. This figure represents the essential components of biological system established between membrane-bound transporters, and the iron cation regulation performed by these transporters situated on membrane boundaries.

*Trichoderma* spp.-derived microbial biostimulants represent another significant category utilized in crop production. These biostimulants enhance plant nutrient levels and resilience to environmental stressors by stimulating root growth and facilitating iron uptake through the activation of ferritin genes (Figure 2). They also promote nutrient absorption and synthesize auxins and secondary metabolites such as peptides and volatile organic compounds (Vinale et al., 2013; Ahmad et al., 2015; López-Bucio et al., 2015; Fiorentino et al., 2018). On the other hand, a number of different fungi have shown biostimulatory activity on crops, boosting plant growth, yield, and oxidative stress response (Drobek et al., 2019). The presence of nitrogen, sulfur, phosphorus, and other essential elements in soil is regulated by various enzymes released by microbes and plants forming a complex system of plant-soil-microbe interaction. The enzymes of this interaction include acid and alkaline phosphatase, sulfur, phosphorus dehydrogenases, and urease activity (Kompała-Bąba et al., 2021).

**Figure 3:**
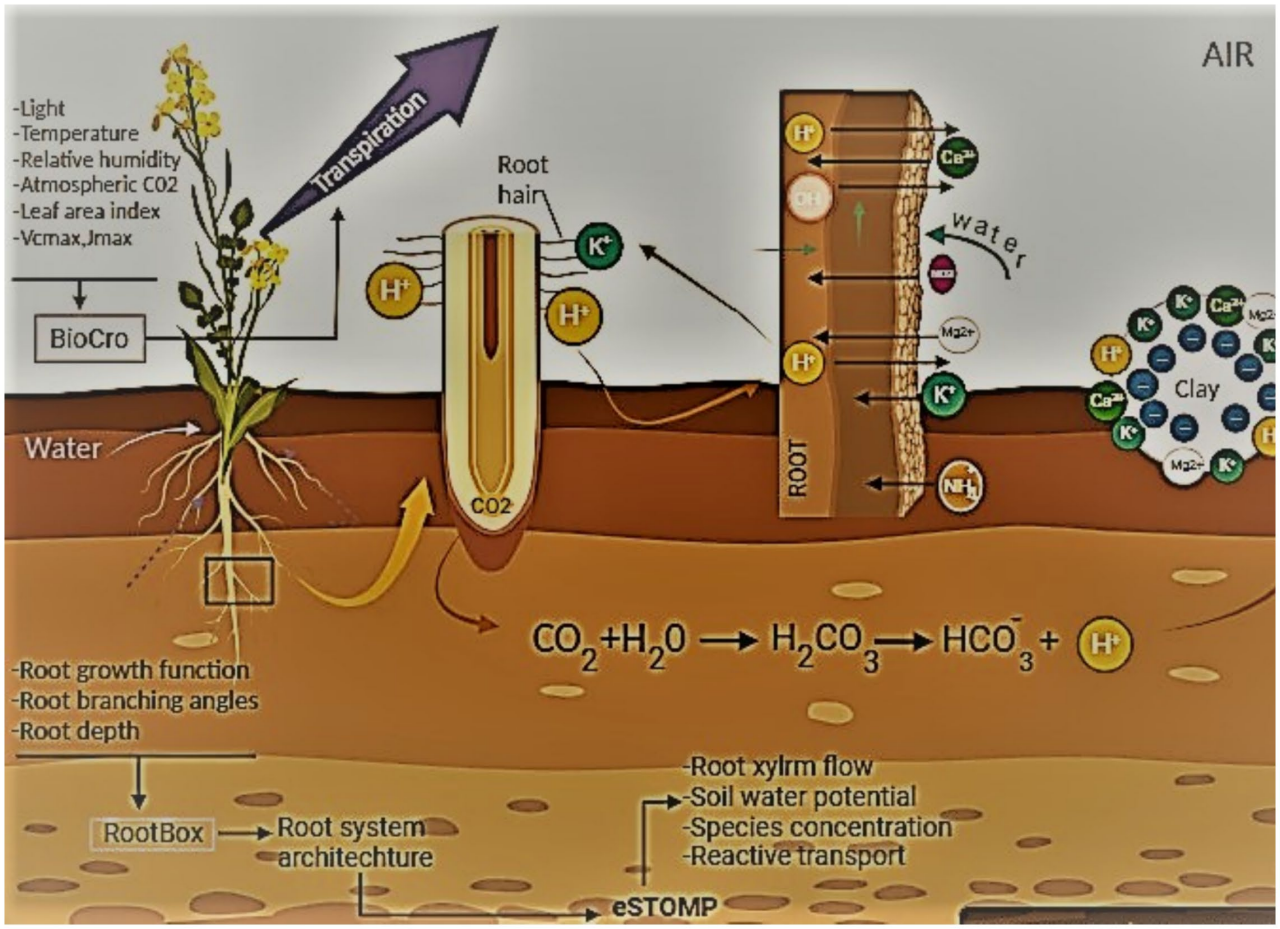
Role of humic materials in increasing soil’s cation exchange capacity involves interaction between root membrane transporters and minerals and increases soil’s cation exchange capacity. This figure illustrates the interactions going on at the level of soil and plant roots, where elements of root biology govern the biochemical interactions with soil’s microenvironment.

On the other hand, despite their high biodiversity, very few plant growth-promoting bacteria (PGPB) are utilized in a variety of formulations (Olivares et al., 2017). Their actions may enhance tolerance to biotic stressors by inducing systemic tolerance, synthesizing volatile organic compounds, and leveraging the presence of PGPB (Kloepper et al., 2004; Glick, 2014; Ruzzi and Aroca, 2015; Bertrand et al., 2021). The primary negative effects that abiotic stressors (such as salinity, drought, and heat) have on plants are alterations in the equilibrium of endogenous hormones (such as the production of ethylene, an increase in abscisic acid, and a decrease in cytokinin levels). As a result, the regulation of plant homeostasis necessitates a reduction in the growth of shoot and root (Yang et al., 2009). Because they encourage the production of indole acetic acid (IAA)—a natural auxin phytohormone—which, in turn, alters the architecture of the roots and causes them to develop, PGPB play a role at this crucial point, which results in a larger root area and more root tips. More meticulously, these extra (exogenous) phytohormones and the endogenous chemicals currently present in plant tissues manage cell multiplication, especially in the roots, working with the uptake of water and minerals from the dirt, which are important for plant development. Roots and shoots can communicate with one another through hormonal signaling by passing endogenous hormones from the xylem to the shoots, which act as hormonal sinks (Wang and Irving, 2011). In fact, the aerial parts of the plant are controlled by the roots. However, a number of additional phytohormones related to the soil microbiome have been discovered in the root-soil environment, in addition to the hormones that the plant may produce internally (Lu et al., 2021). These phytohormones might enter the plant through the xylem flow and manage plant development in light of their equilibrium. The soil microbiome, for instance bacteria and fungi, may also produce phytohormones, which serve as signals for root function (Lu et al., 2022). Plant roots may also produce phytohormones (Rosier et al., 2018). The equilibrium of these *ex-planta* hormones, which also interact with internal plant hormones to govern plant growth and development, is controlled by the synthesis and absorption of the roots, along with the synthesis, absorption, and degradation of hormones by soil microorganisms (Yang et al., 2009; Lu et al., 2021).

Investigation has proposed that substances resembling humic compounds, such as humic and fulvic acids, might exhibit biostimulant characteristics due to their capacity to imitate auxin and cytokinin (Pizzeghello et al., 2013; De Pascale et al., 2018; Puglisi et al., 2018). In general, humic compounds primarily enhance fruit quality and resilience to abiotic stress, promote root growth and morphology, boost nutrient uptake and nitrogen use efficiency (NUE), and enhance overall crop performance (De Hita et al., 2019; Vujinović et al., 2020). The kind of soil and the organic matter present in rhizosphere defines the rate of flow for movement of nutrients and humic acid desired by plants. This creates a synergism between the presence of bioactive compounds and plant growth. Auxins and other precursors are released when humic and fulvic acids are broken down, so these acids may also have hormone-like effects on plant growth (Olaetxea et al., 2018). The release of biomolecules resembling auxin suggested the hormone-like effect of the humic acid on tomato plants, a classical case of biological mimicry, wherein a metabolite having different functional groups represents another metabolite with in-original functional group (Jindo et al., 2012; Pizzeghello et al., 2013; Conselvan et al., 2018; Lucini et al., 2020). Due to the fact that humic-like substances and humic acids in particular can be obtained from a variety of unrefined components, such as regular natural matter, plant tissues, and biowaste, and they have varying effects based on their subatomic weight (Canellas et al., 2015; Scaglia et al., 2017).

Biopolymers such as chitosan, which have been used in a number of applications on horticultural crops, and the biostimulant properties of phosphate (Phi) were also reported (Gómez-Merino and Cand-Trejo-Téllez, 2015; Pichyangkura and Chadchawan, 2015). Regarding Phi, it is used as a fungicide and an effective microcin that also serves as a beneficial additive to increase both yield and nutritional status. On the other hand, it brings growth-promoting properties, such as better root growth and increased uptake and assimilation of nutrients (Rickard, 2000; Gómez-Merino and Cand-Trejo-Téllez, 2015). On the other hand, and due to its intriguing effects on crops, chitosan, a biopolymer made by deacetylating chitin, has been the subject of intense research in recent years. It is produced commercially from sea food shells, and its primary use is in plant defense against pathogens due to its potential to induce the production of pathogen-protective molecules (Pichyangkura and Chadchawan, 2015).

### 2.2 Classification benefits attributed to PB

Biostimulants have also been reported to have increased photosynthetic activity, anti-oxidant enzymatic activity, drought, salinity, and stress tolerance (Singh, 2016). However, the available products can vary significantly because of composition of chitosan. It is a biopolymer characterized by differing levels of deacetylation and polymerization. As a result, these variations may lead to diverse effects on crops (Malerba and Cerana, 2018). The biostimulant industry is actively exploring waste materials and by-products with significant biological potential, potentially introducing a new category that offers alternative approaches for managing by-products and complementing existing categories (Ugolini et al., 2015; Pane et al., 2016). In this unique circumstance, new biostimulatory items that might further develop plant growth through an auxin-like method of activity have shown promising outcomes on account of the creation of dissolved organic matter (DOM) through anaerobic processing (Messias et al., 2015). Shale water is produced when pyrobituminous shale rock is pyrolyzed. It has been demonstrated that shale water has significant biostimulant effects on horticulture crops and can be utilized as a biofortifying and yield-enhancing agent. Finally, other substances such as vitamins and melatonin have been shown to have biostimulatory effects particularly when subjected to abiotic stress. These substances not only encourage the biosynthesis of secondary metabolites but they also recover the functionality and quality of the final products (Hanson et al., 2016; Teklić et al., 2021).

### 2.3 Biosafety and ethics

**Figure 4:**
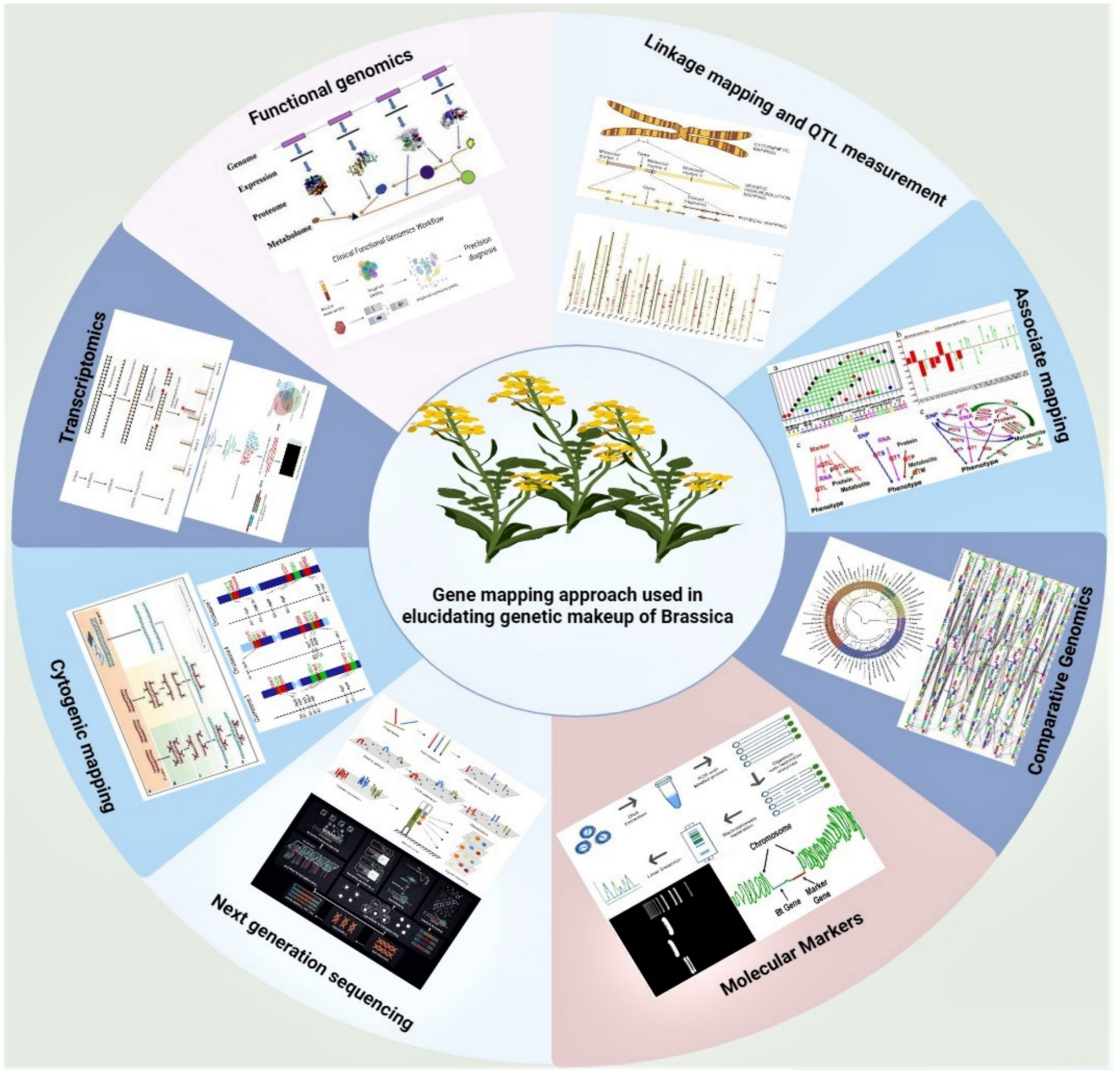
Elements of genetic sequence creation: genetic makeup of Brassicaceae deciphered by molecular approaches. This figure depicts the example of brassica and shows how the gene mapping helps in elucidating the complete molecular biology background of any concerned crop.

The importance of any compound is defined by parameters such as necessity, employed subjectivity, safe to use, acceptance by people, and positive marketing response. Once a compound is demarcated crucial and safe, its response of acceptance by public comes from ethical points. Since no animal-derived product is ensured herein yet, the endangered plant supplementary materials are forbidden for the implementation in PBs (see Figure 4).

Biosafety protocols should prioritize Environmental and Human Safety Indices (EHSI) and bioassays over taxonomic classifications (Vílchez et al., 2016; Barros-Rodríguez et al., 2020), especially given the developing nature of the biostimulant industry and the diverse range of substances and organisms that fall under this category. Additionally, careful consideration should be given to unintended consequences that may contribute to adverse effects on reactive nitrogen losses (Souza et al., 2019). One of the primary research topics for the key area of focus on biostimulant characterization of new substances and compounds is the (1) evaluation of the composition of the biostimulant, (2) production procedures that are uniform (3) the description of plant responses particularly when paired with the conditions of environment, (4) determining how biostimulant-containing products affect particular crops, and (5) fine-tuning of application timing and dosage (Toscano et al., 2021).

### 2.4 Biostimulant-generated chemical interactions in the biological system

#### 2.4.1 Humic substances

Once a living counterpart falls off from its roots of existence and falls onto land, it is marked for decomposition as the current biological environment is oxidizing in nature as opposed to reducing one as that was during early evolution. Once the living organism is dead, it heads for decomposition by virtue of process of oxidation under natural environment. Since the oxidation means the loss of electrons, these electrons from decomposed material when released into soil creates an amorphous precipitate which may vary from black to brown in color. These precipitates originated from dead decomposed organic matter are collectively called as humic substances.

Franz Karl Achard noted solid HS formation while mixing a relatively basic material KOH (potassium hydroxide) in peat and extracting it with precipitated decomposed biomass that was naturally formed. This made the pivotal basis of preparing the alkaline extract from the soil. Johns Jackob Berzelius noted the aquatic HS formation from spring water. Moreover, the liquid and solid HS are natural compounds derived from decomposed biomass. However, basic functional groups such as phenol, carboxyl, and ester-related linkages are common to both types. The nomenclature of HS is defined on the basis of insolubility at low hydrogen ion concentration, whereby HS becomes humic acid. Now, the humic acid is the biological accepted version of HS, governing important nutrient uptake and exchange within soil-plant-water ecosystem. More precisely quoting for humic acids, the microbial degradation and atmospheric oxidation of dead organic matter (in-essential) composed of variable fractions of cellulose (most abundant homopolysaccharide), lignins, and charcoal that give rise to humic acid. Several instances are discussed here which ascertain the enhanced nutrition-based growth of plants (Trevisan et al., 2010; Zanin et al., 2019).

##### 2.4.1.1 Humic acid

- Anti-oxidant activity of any metabolite is representative of its extent of its ability to scavenge free radicals. Humic acid promotes the anti-oxidant activity in *Achillea millefolium L*., stimulating its content of phenols and flavonols and making it somewhat more tolerant to pathogens. Moreover, it enhances photosynthetic pigment biosynthesis, meaning that plants reside in its basic physiological parameters such as roots, leaves, flowers, and shoots. It is also attributed to fulvic acid (another derivative of HS but soluble at all scales of pH in water) (Bayat et al., 2021).
- Genetically, framing the exons by governing the regulation of protein bodies involved for acclimatization of plants induced by heat stress conditions is also another attributed functional scale of action of humic acid and HS. The case of *Arabidopsis thaliana* (Thale cress grass; model organism for plant studies) is peculiar, wherein in response to HS, the concentrations of additional proteins involved in cell trafficking, division, folding, degradation, reactions to heat and inorganic substances, and the cell wall increased, simultaneously reducing centralized distribution of carbohydrates and amino acids (Roomi et al., 2018; Cha et al., 2020).
- HS favored the higher yield of *Brassica napus* (a plant from family Brassicaceae with 372 genera), improved plant net photosynthesis, gas exchange rate, electron transport flux, oil quality, and gas exchange rate, and declined in linoleic, erucic corrosive, and solvent sugars (Lotfi et al., 2018; Nasiri et al., 2023). These all physiological parameters are devoid of *in-planta* action, where HS are found absent. Similarly, in another abiotic stress, such as under drought stress, *Capsicum annuum L*. rapidly decreased leaf stomatal conductance and transpiration rates while simultaneously increasing plant biomass and enhancing root development and photosynthesis as a result of an increase in the amount of chlorophyll (Qin and Leskovar, 2018). All these are attributed by the presence of fulvic and humic acids according to the changing value of pH of water.
- Proline is the amino acid known to be a marker for all types of biotic and abiotic stresses, and HS stimulate its concentration by activating the enzyme cofactors involved in its biosynthesis. Plant development was observed when studied under dry season pressure by *Echinacea purpurea L*., an expanded centralization of proline. Moreover, an enmarked increase in the content of phenols, flavonoids, total chlorophyll values was observed (Khorasaninejad et al., 2018).
- Providing with adequate increase in the amount of photosynthetic machinery, HS stimulate hardiness in the plant growth and yield of *Hordeum vulgare L.* (Abdelaal et al., 2018).
- *Rhododendron* spp. is a plant from native Himalayan ranges. A plant growing at this height of >1,200 meters is prone to oxidative damage by the environment. Moreover, chilling and freezing are commonly noticeable in such areas. HS-supplied enrichment is responsible in this plant for an increase in peroxidase genes (POD1) (Elmongy et al., 2020).
- Plant biomass and tuber yield are both increased by *Solanum tuberosum* and further created plant advancement, supplement transport, and photosynthetic limits under dry season tension, as attributed to the presence of HS (Man-hong et al., 2020).
- *Zea mays* makes nitrogen and water work better as it is a C_4_ plant; the upregulation of qualities that are related to nitrate carriers, supplement retention, and the development of water is enhanced due to HS and humic acid (de Azevedo et al., 2019).

##### 2.4.1.2 Fulvic acid

The important difference between humic and fulvic acids is that former is insoluble at low pH in water while the latter is soluble at all pH scales. The beneficial aspects derived from these two are the same except the fact that certain nutrients become bioavailable at a specified pH. Moreover, nature has compromised the nutrient availability to plant roots by creating a pH barrier, beyond which certain nutrients are accessible and non-accessible by plant metabolic molecular machinery. Prominent examples exist where the role of fulvic acid is largely involved in regulation aspects. Some examples are presented here:

- Improved germination parameters of *Beta vulgaris* alongside increased quantity of soluble sugar, root size, and yield (Braziene et al., 2021).
- By regulating genes related to ascorbate, glutathione, and flavonol biosynthesis, *Camellia sinensis L*. increased the antioxidant defense against water stress and the amount of chlorophyll and water in the leaf and reduced the ROS production in part of the supplementation by fulvic acid (Sun et al., 2020).
- Long withstanding agility and ability to harsh climate can overcome by *Hordeum vulgare* due to the presence of essence of fulvic acids (Braziene et al., 2021).
- Up-regulation of qualities related to early nodulation flagging, nitrogen digestion, supplement carriers, and hydrolases by *Medicago sativa*, increasing plant yield and biomass (Capstaff et al., 2020).

#### 2.4.2 Protein hydrolysates

Plant biostimulants known as protein hydrolysates (PH) consist of a blend of peptides and amino acids, yielding positive outcomes in plant productivity (Colla et al., 2015). The PH can be classified according to their sources, originating from either animal material or plant material. Their impact is influenced by both their source and the physicochemical or enzymatic processes used to produce them (Colla et al., 2014). Animal protein sources primarily originated from animal by-products, such as epithelial tissue, collagen, feathers, leather, and fish remnants, while plant protein sources are obtained from plant residues such as alfalfa, seeds, legumes, and hay (Schiavon et al., 2008; du Jardin, 2015). PH sourced from animals predominantly contain a blend of free amino acids and peptides (Ertani et al., 2009; Colla et al., 2015). Some examples given below suggest the role PH in nature.

- Under dry season stress, *Brassica oleracea* expanded its photosynthetic rate and stomatal conductance and decreased in the negative effects of stress on gas exchange and transpiration rates Kałuzewicz et al., 2017) (as a result of induction from PH).
- *Diplotaxis tenuifolia L*. enhances biomass, chlorophyll biosynthesis, and plant yield with an increased photosynthetic rate and antioxidant activity in the leaves and also increased levels of nutrients and organic acids (Caruso et al., 2020) responded in positive sense to PH.
- The application of vegetal-derived bioactive compounds promoted the microbiome biodiversity in Lettuce, resulting in increases in plant biomass and leaf chlorophyll (Luziatelli et al., 2019).
- Stomatal conductance and photosynthetic rate were both increased by *Olea europaea*, increased plant growth and biomass, and emphatically affected the sink-to-source proportion (Almadi et al., 2020).
- In *Solanum lycopersicon* L., the application of (biostimulant/PH) resulted in enhanced plant growth parameters such as biomass, chlorophyll content, and phenolic content. Additionally, there was an increase in root growth, photosynthetic rate, solvent sugar fixation, antioxidant activity, and nutrient uptake, along with improvements in primary metabolisms. Under drought stress conditions, treated plants exhibited increased biomass, transpiration rates, and stomatal conductance. Furthermore, the treatment helped maintain redox status, protected against oxidative stress caused by reactive oxygen species (ROS), and mitigated the accumulation of cytokines, jasmonic acid, indole-3-acetic acid (IAA), and antioxidants (Ertani et al., 2017; Michaletti et al., 2018; Sestili et al., 2018; Casadesús et al., 2019; Paul et al., 2019; Lucini et al., 2020).

#### 2.4.3 Sea weed extracts

Prominent examples of sea weed extracts (SWE) are given below:

- *Ascophyllum nodosum* significantly enhances the ability of *Arabidopsis thaliana* to withstand oxidative stress and drought by reducing the accumulation of reactive oxygen species (ROS) and cellular damage. This is achieved through the downregulation of genes linked to stress-induced growth impairment while simultaneously boosting ROS scavengers, cell cycle, and division processes (Omidbakhshfard et al., 2019; Rasul et al., 2021; Staykov et al., 2021).
- Under drought stress, *Glycine max L*. increased chlorophyll content, antioxidant activity, photosynthetic activity, and efficiency and enhanced production and root growth through photo assimilation in response to *Echinochloa maxima* (do Rosário Rosa et al., 2021) under the stimulation of seaweeds.
- Multiple seaweed extracts induced an increase in the length of pollen tubes *Solanum melongena L*., as a result of rapidly fertilized ovules, which resulted in improved plant fruiting and flowering (Gavelienė et al., 2021).
- Plant biomass, protein, nutrient, and leaf phenolic compound concentrations are all increased in *Spinacia oleracea L*., with reported rate of increase in photosynthesis and production of chlorophyll and enhanced nutritional value (Rouphael et al., 2018a,b).

#### 2.4.4 Microorganisms: plant growth-promoting rhizobacteria

Prominent examples of microorganisms, especially PGPR, are as follows:

- The leaves of *Amaranthus hybridus L*. had a higher concentration of nutrients, improved photosynthetic pigments, nutritional quality, and plant growth under PGPR influence in rhizosphere (Ngoroyemoto et al., 2020).
- PGPRs when coexisting with *Brassica napus* contributed to increased seed fatty acid content, augmented plant biomass, improved yield parameters, and overall plant growth (Jiménez et al., 2020).
- *Brassica rapa* L. increased plant biomass, number of leaves, root length, and total IAA, P, and N contents (Widawati and Suliasih, 2020) in response to PGPR.
- Chlorophyll and NPK contents, plant biomass, and yield were undeniably expanded in *Cicer arietinum L*. in response to PGPR (Rafique et al., 2021).

## 3 Broad marketing scenario of PBs

As per the latest version drafted by the European Commission, non-microbial organic plant biostimulants encompass natural elements such as humic acid (HA), protein hydrolysates (PH), and seaweed extracts (SWE). The SWE segment controls 37% of the market, while the first two categories control half of the market. Humic substances such as humic and fulvic acids are naturally occurring organic molecules that are the result of the biological and chemical transformations of dead organic matter (Nardi et al., 2016). Most of the times, the humic substances are applied as soil soak but occasionally (fulvic acids) are applied as foliar application. According to du Jardin (2015), the chemical, physical, and biological properties of soils are influenced by humic substances, which have long been considered essential components of soil structure and fertility. The biostimulation effect of HAs on soil nutrient availability and uptake has been linked to a number of mechanisms that affect plant physiology and soil processes: (i) enhancing the structure of the soil, (ii) neutralizing the pH of the soil, (iii) enhancing the phosphorus solubility by interfering with calcium-phosphate precipitation and preventing leaching, (iv) triggering plasma membrane H^+^ ATPase activity, (v) enhancing lateral root induction and hair growth, and (vi) stimulating nitrate assimilation by upregulating the target enzymes (Delgado et al., 2002; García-Mina et al., 2004; Schmidt et al., 2007). According to du Jardin (2015), the biostimulatory action of HAs is significantly influenced by the conditions of soil fertility, with HAs performing better in soils with low organic matter content and low fertility (De Pascale et al., 2018). The wellspring with higher plant execution in light of HAs separated from humidified natural matter (e.g., peat), fertilizers, and vermicomposts instead of those approaching from fossil humus is another factor that contributes to the changeability in the effects of HAs. In addition to their influence on plant metabolism and physiology, both directly and indirectly, numerous studies have indicated the biostimulant properties of humic acids (HAs), particularly their ability to provide stress protection, notably against salinity and drought (García et al., 2012; Petrozza et al., 2014). Salt tolerance and drought tolerance may be mediated by reducing lipid peroxidation and hydrogen peroxide, increasing the amount of proline, and regulating gene expression in different ways, enhancing the chemical, microbiological, and physical properties of the soil and root growth (Calvo et al., 2014).

According to research claims, plant- and animal-based PH, which are composed of a mixture of free amino acids and oligopeptides and polypeptides that serve as signaling molecules, make up a significant subset of organic non-microbial PBs. PHs are primarily used as foliar sprays, but they can also be used to treat seeds and drench substrates (Colla et al., 2015). In various studies conducted in both greenhouse and open field settings, PHs demonstrated notable effectiveness as plant biostimulants (PBs), triggering physiological and molecular mechanisms that enhance growth and productivity (Colla et al., 2017). The following factors directly affect the biostimulation activity and abiotic stress tolerance of PHs: key proteins engaged with N absorption (NR, NiR, GS, and GOCAT) and C digestion are set off, as are auxin- and gibberellin-like exercises, cancer prevention agent protein movement, shade biosynthesis, auxiliary metabolite creation, citrate synthase, malate, and isocitrate dehydrogenase (Schiavon et al., 2008; Ertani et al., 2009, 2017; Rouphael et al., 2017a, 2018a,b; Sestili et al., 2018). In addition to their direct impact, applying PHs as foliar sprays or substrate drenches also resulted in indirect effects on crop performance and nutritional status (Colla et al., 2014). Indeed, the utilization of PHs has led to enhanced nutrient uptake by expanding the effective soil volume utilized by the root system, notably through the augmentation of root hair diameter, density, and length (Colla et al., 2015).

Further investigation is required to fully understand the mechanisms and modes of action of organic non-microbial plant biostimulants, despite notable advancements in this field. This entails standardizing the raw materials, properties, and extraction techniques and determining the optimal timing, method, and dosage for application under various species and environmental conditions.

## 4 Advancements in next-generation sequencing

### 4.1 Components in a genetic sequence development

The genetic material of the plant cell is present inside the nucleus, is accessible by transcriptional and translational machinery of the cell, and is comprised of enzymes and other factors (proteins, carbohydrates, and lipids). Most of the coded information of the cell is displayed by a set of mRNAs (messenger Ribonucleic acid) in genetic elements called codons that ultimately and exclusively give rise to proteins only. However, genes concerned for glycobiology (study of carbohydrates) and lipidome (overall lipid fraction in a cell) are assigned to their function with protein counterparts, whose successive addition and deletion are regulated by a set of regulatory enzymes and genes concerned with the, and this creates a system of cell’s function in relation to the environment that it perceives. The coordination among different chemical metabolites as a result of gene expression is displayed as a phenotypic expression. This journey of genotypic to phenotypic expression is called systems biology. Although dynamic in action, the equilibrium is well attained in terms of lipidomics, transcriptomics, proteomics, phenomics and genomics to govern a well-destined living function in terms of systems biology (Breitling, 2010) (Figure 4).

There are several steps in which a way is designed to assign a gene its function, which is scientifically called as annotation. Gene annotation (to assign a gene its function) is a solo feature behind all sophisticated techniques, which utilize the information coded in base pairs in a stretch of nucleotide. To begin with the isolation of a gene, it has been advanced to a level where nanograms to attograms of DNA (deoxyribose nucleic acid) can be quantified by modern day polymerase chain reaction (PCR). Nonetheless, primers are available at biotechnology product synthesizing industry; the respective amplification is thought to be illuminated in the presence of fluorescent and non-fluorescent markers. However, certain gaps remain like whether PCR amplification is limited to a certain region of the whole gene or certain important regulatory elements are missing from a PCR product that would be the ultimate determinants of gene action and thus functional gene annotation. Gene cloning through vectors has shown us remarkable opportunities to create multiple copies of gene facets and their products. However, to go to the extent of demarcating the single point of gene action, the proper nucleotide base pair number, their relative position, and their length are needed to be met to ascertain specific gene annotation. The true criterion is to count of the number of base pairs and the area that they span the genome through base pair locations. These base pair locations are the sites from where nucleotides are counted in a direction ahead. The allotment of any of four base pairs like one after the other creates a specific sequence in which they are either repeated, non-repeated, overlapped, non-overlapped or may contain a stretch already with defined set of nucleotides. For all the sequencing technologies developed so far, the basic aim is to allocate the sequence of each nucleotide one after the other and generate a sequence, in which it is shown which nucleotide base pair follows the other. This has been achieved with chemical sequencing known as pen-name Maxam and Gilbert sequencing and Sanger sequencing (devised after the name of their inventors). Now, once the locations within a genome have been framed for base pairs, it is not like that a continuous stretch is likely to originate. The random base pairing, overlapping, and deletion (due to technical errors) are the problems commonly encountered while sequencing a fragment of genome. Modern day sequencing or, quite clearly, next-generation sequencing overcomes these limitations, but, to a particular point called sequence depth, segments of base pairs are unknown. The segments initialized for sequencing are aligned by creating a library, called gene library. In this gene library, the overall segments of a gene (intron and exon) from which nucleotides are extracted in base pairs and laid onto a platform of gel runs, followed by automated analyzers, define nucleotide (kind and type) on the basis of its resemblance to the structural output of a gene (Heather and Chain, 2016).

The need of hour is the more sophisticated sequence analyzers which can define sequence depth in an extendable manner. However, evident from current case scenarios is the fact that larger and pure gene library constructs are needed for successful sequencing of gene as the cross-contaminants are hindering agents in successful true-to-sequence generation.

The major focus has been on advancements concerned within and through these sequencing technologies, which have created guiding tools for any biological in-gene action metabolite function and biosynthesis. With biosynthesis, the metabolic pathway which already contains a set/series of enzymatic steps is controlled by in-gene action. Moreover, data are needed to be generated for successful gene annotation for each and every metabolite concerned. Agricultural production is often limited by the presence/absence of crucial metabolites, which ultimately define the quality parameter of a product. Glucosinolates and phytic acid in mustard are important examples whose elevated and lowered concentrations due to in-gene action actually define the type and palatability of different genotypes and varieties of mustard (Das et al., 2022).

### 4.2 Sequencing strategies

The time period during which a particular sequencing technology has been invented is given a generation factor. Old sequencing technologies are tended to be summarized in first generation. Quite-a-new sequencing technologies are included in second generation. Relatively-new sequencing technologies are included in third generation and latest sequencing technologies are being put together in fourth generation sequencing technologies. The complete technical details of sequencing techniques concerned are far beyond the scope of present context, but an overall scale of technical implications has been included in the text. Starting from first generation, second generation, third, and fourth generation sequencing techniques, technical implications are being included here. Maxam–Gilbert and Sanger sequencing (also called di-deoxychain termination method) which are being used from 1980s onward are basic techniques with which sequencing progressed into a varsity of version.

#### 4.2.1 Techniques concerned for second generation sequencing

##### 4.2.1.1 454 sequencing or pyrosequencing

Pyrosequencing was first described in 1993 by Nyrén et al. (1993) who used streptavidin-coated magnetic beads, recombinant DNA polymerase devoid of 3′ to 5′ exonuclease activity (for proofreading), and luminescence detection with the firefly luciferase enzyme. To sequence single-stranded DNA, a mixture of three enzymes (DNA polymerase, ATP sulfurylase, and firefly derived luciferase) and a nucleotide (dNTP) is added. The incorporation of the nucleotide is controlled by measuring the light that is emitted. The incorporation factor for nucleotides, which indicates the number of complementary nucleotides on the template strand, is determined by the intensity of the light. Before the subsequent nucleotide mixture is added, the previous nucleotide mixture is withdrawn. This procedure is meant to be repeated with each of the four nucleotides until the DNA sequence of the single-stranded template is determined.

##### 4.2.1.2 Illumina (Solexa): HiSeq and MiSeq sequencing

In 2006, Solexa Genome Analyzer was purchased and was available for sale in 2007 (Shendure et al., 2005). Before, it is observed as the best sequencing system ensuring >70% strength of the market, particularly with the HiSeq and MiSeq stages. With removable fluorescently labeled chain-terminating nucleotides, the Illumina sequencer, in contrast to Roche 454, produces a more cost-effective output than Roche 454 (Margulies et al., 2005; Metzker, 2010). By PCR span enhancement, called group age, into small provinces known as polonies, the clonally improved layout DNA for sequencing is made (Shendure and Ji, 2008). Sequencing data produce more data per run than other systems (600 Gb, Gigabites), more limited read lengths (approximately 100 bp; base pair), lower expenses, and run times that are essentially longer (3 to 10 days) (Liu et al., 2012). The Next Seq 500, HiSeq series 2500, 3000, and 4000, and HiSeq X series 5 and 10 with mid-to-high output (120–1,500 Gb), as well as the MiSeq, a small laboratory sequencer with outputs ranging from 0.3 to 15 Gb and turnover rates suitable for targeted clinical and small laboratory applications, are a few of the sequencing machines that Illumina offers.^1^ While the top of the line machines utilize the equivalent sequencing and Polony innovation, the MiSeq can deliver sequencing, which brings approximately 1 to 2 days (Liu et al., 2012). The most recent strategy of Illumina to manufacture long per-use application made the TruSeq nano resolving complex retro-transposably evident (McCoy et al., 2014).

##### 4.2.1.3 SOLiD sequencing

Sequencing ligation is used in the Applied Biosystems SOLiD technology, which is now a Life Technologies brand. This method labels a pool of all possible oligonucleotides of a particular length in accordance with the position of the sequence. Annealing and ligation are conducted to oligonucleotides. Whenever the matching sequences are found, the DNA ligase enzyme performs ligation, and a signal indicating the nucleotide at that position is produced. Before the DNA is sequenced, amplification is performed with emulsion PCR. As a result, beads with one copy of the same DNA molecule are placed on a glass slide (Valouev et al., 2008). Amounts and lengths of the subsequent successions are similar to Illumina sequencing. It has been reported that sequencing palindromic sequences by ligation presents some challenges (Huang et al., 2012).

##### 4.2.1.4 Polony sequencing

Polony, the first high-throughput sequencing system, was created at the Harvard George M. Church Laboratory. It was used to sequence the entire *E. coli* genome in 2005. It sequenced an *E. coli* genome utilizing a computerized magnifying lens, ligation-based sequencing science, an *in vitro* matched label library, emulsion PCR, and ligation-based sequencing science at an expense roughly one-tenth that of Sanger sequencing (Shendure et al., 2004). The advancement was approved to Agencourt Biosciences, which was appropriately transformed into Agencourt Individual Genomics and, in the end, included in the Applied Biosystems Strong Stage. Life Advancements, which is currently a division of Thermo Fisher Scientific, joined the project after Applied Biosystems.

##### 4.2.1.5 Massively parallel signature sequencing

In the 1990s, Sydney Brenner and Sam Eletr’s Lynx Therapeutics company developed massively parallel signature sequencing (or MPSS), a novel approach to high-throughput sequencing technologies. Bead-based MPSS reads the sequence in four-nucleotide increments using a complicated strategy of adapter ligation and adapter decoding. It was therefore vulnerable to sequence-specific bias or the loss of particular sequences. Lynx Therapeutics only performed MPSS “in-house” due to the complexity of the technology, and no DNA sequencing machines were sold to other laboratories. A partnership was formed in 2004 between Lynx Therapeutics and Solexa, which was later acquired by Illumina. This resulted in the creation of sequencing-by-synthesis, a more straightforward approach that was acquired from Manteia Predictive Medicine and rendered MPSS obsolete. The fundamental features of the MPSS output, which included hundreds of thousands of brief DNA sequences, were typical of subsequent high-throughput data types. These were utilized to the arrangement of cDNA (reciprocal DNA) for estimating levels of quality articulation in the MPSS study (Brenner et al., 2000).

#### 4.2.2 Techniques concerned for third generation sequencing

##### 4.2.2.1 Single molecule real time sequencing biosciences

The PacBio RS II sequencer and the SMRT ongoing sequencing framework are exchanged at Pacific Biosciences (Schadt et al., 2010). SMRT cells require 150,000 ultra-microwells at a zeptoliter to perform SMRT sequencing on a 10–21 scale, where a DNA polymerase enzyme-embedded particle is immobilized at the bottom of each well using a biotin-streptavidin framework in zero-mode waveguide (ZMW) nanostructures (biotin-streptavidin interactions are strongest non-covalent biochemical interactions). Nucleotides are added to the growing strand after fluorescently labeled dNTP analogs are incorporated into the single-stranded DNA template by immobilized DNA polymerase. Charged coupled device (CCD) cameras continuously detect the 150,000 ZMWs as a collection of distinct pulses that are transformed into the template sequences. Because all four nucleotides are added simultaneously and measured in real time, the speed of sequencing is significantly faster than that of technologies, in which individual nucleotides are flushed sequentially. Additionally, the read lengths were approximately 900 bp, and the initial accuracy was 99.31% (Metzker, 2010). Longer reads with an accuracy of more than 99.99% were obtained by employing SMRT bell templates, which circularized and sequenced the template multiple times (Travers et al., 2010; Koren et al., 2013). Once sequencing begins, the computational blade center of the system makes use of real-time signal processing, base calling, and quality assessment. The primary analysis software sends the data directly to secondary analysis software, SMRT Analysis, which can process sequencing data in real time. Read-length, distribution, polymerase speed, and quality measurement are all included in this data. Similarly, the optional examination instruments contain a full set-up of devices to investigate single-particle sequencing information for different applications.

##### 4.2.2.2 Heliscope sequencing

The Helicos sequencing system, which Helicos Biosciences sold, was the first commercial implementation of single-molecule fluorescent sequencing (Shendure and Ji, 2008). The sequencing provider SeqLL currently sequences genomic DNA and RNA using the Helicos sequencing system and the Heliscope single-molecule sequencers. Cleavage of DNA is attained, followed by the addition of synthetic poly A sequences (in this case called tailing, as poly A sequences are attached at 3′ ends) and finally hybridization to the surface of flow cell, achieving massive (billions of molecules) parallel sequencing. The oligo-dT50 bound on the outer layer of expendable glass stream cells is straightforwardly joined to the poly A-followed sections of DNA particles. When a picture of one nucleotide for every DNA grouping has been caught, the recurrent cycle is come by the joining of fluorescent nucleotides with an ending nucleotide. The procedure is then repeated until the fragments are fully sequenced (Eid et al., 2009; Travers et al., 2010). The sequencing system combines DNA polymerase-based synthesis sequencing with hybridization sequencing (Fuller et al., 2009).

The G + C (Guanine + Cytosine) content and size biases that have been observed in other technologies are virtually eliminated because sample preparation does not involve ligation or PCR amplification The Heliscope sequencing reads have a mean length of 35 bases and range from 25 to more than 60 bases. Sequenced RNA creates quantitative transcriptomes of cell and tissues (Hart et al., 2010).

#### 4.2.3 Techniques concerned for fourth generation sequencing

##### 4.2.3.1 DNA nanoball sequencing

High-throughput DNA nanoball sequencing was used to help determine an organism’s complete genomic sequence. The organization named Complete Genomics used this method to grouping tests presented by various scientists. Using rolling circle replication, small fragments of genomic DNA were amplified into DNA nanoballs. As a result, ligation-unchained sequencing was used to determine the nucleotide sequence (Drmanac et al., 2010). This method of DNA sequencing, as stated by Porreca et al. (2006), enables the sequencing of a large number of DNA Nano balls per run and is more cost-effective than other high-throughput sequencing platforms. Although each DNA nanoball only produces short DNA sequences, mapping the short reads to a reference genome is challenging. Numerous genome sequencing projects have used this innovation, and extra purposes are expected (Xu et al., 2019).

##### 4.2.3.2 Nanopore DNA sequencing

DNA is transformed into an ion current by the method as it moves through the nanopore. This change is caused by the shape, size, and length of the DNA sequence. Each type of nucleotide prevents particles from moving through the pore for a different amount of time. Modified nucleotides are not required because the procedure is carried out in real time. Nanopore sequencing is also known as “long-read” or “third-generation” sequencing, just like SMRT sequencing. In the beginning, this method was based on exonuclease sequencing, in which electrical signals from nucleotides passing through alpha-hemolysin pores were covalently bound with cyclodextrin (Clarke et al., 2009). The current focus areas of nanopore sequencing are protein-based nanopore sequencing and solid state nanopore sequencing. Film protein structures such as hemolysin and MspA (*Mycobacterium smegmatis* Porin A) are used in protein nanopore sequencing, and their ability to distinguish between individuals and groups of nucleotides is extremely promising (Dela-Torre et al., 2012). In contrast, synthetic materials such as silicon nitride and aluminum oxide are used in solid-state nanopore sequencing due to their superior mechanical strength and chemical and thermal stability (Pathak et al., 2012). For this version of sequencing, a fabrication method is required because the nanopore array can contain hundreds of pores with diameters of below 8 μm.

The above described methods of sequencing the DNA are obliged to sophisticated instrumentation and require protective experienced handling. Table 2 shows the forums associated with next-generation sequencing technologies. Although most of the steps are automated, isolation of DNA, creation of library, constructs, and sample injection are key tasks to be performed before any sequencing analysis. The real-time analysis of PCR amplified products along with concomitant gene expression reveals the fact that all important metabolites are biosynthesized not all the times (Table 3). Their expression level varies with time according to environmental setup assigned to the cell by nature. Moreover, to quantify these types of metabolites, the important responsible genes (called candidate genes) are monitored throughout the isolation and sequencing steps for plant biostimulatory studies.

**Table 2:** Next-generation sequencing forums and platforms.

S. No.	Next generation sequencing forum	Platform	Platform characters/reads	Generation
1.	Ligation sequencing	SOLiD	>50 bp; <80 bp	2nd
2.	Synthesis sequencing	454 Pyro-Sequencing Illumina Sequencing Ion Torrent	<1,000 bp <100 kbp <400 bp	2nd
3.	Single molecule long read	Bioscience (Pacific) Oxford Nanopore	<200 kbp Precise range (8–20 kbp)	4th

**Table 3:** Tissue time-specific expression of genes in plant/plant tissue in relation to metabolite production.

S. No.	Plant/plant tissue	Metabolite	Time	Reference
1.	*Hordeum vulgare*	Glycerophospholipids & polyketides Chlorogenic acid & hyrdocinnamic acid	Seed germination 3-leaf stage	Gupta et al. (2019) Piasecka et al. (2017)
2.	*Oryzae sativa*	Isoleucine, phenylalanine & spermidine Lutein & beta-carotene	Flowering Maturation stage	Roessner et al. (2006) and Zarei et al. (2018)
3.	*Triticum aestivum*	Ubiquinone, guanine & chorismate Phenolamides & polyphenols	Mature leaves Mature kernels	Matthews et al. (2012) and Michaletti et al. (2018)
3.	*Zea mays*	Asparagine, glutamic acid & inositol Anthocyanins & methoxylated flavonoids	8-month old kernel Mature kernel	Barros et al. (2010) Jin et al. (2017)
4.	*Sorghum bicolor*	Cyanogenic glycosides	4-leaf stage	Mareya et al. (2019)

To target the candidate genes, at precise gene locus (the position where a particular known segment of gene lies within the DNA segment), CRISPR/Cas9 technology has paved the fruitful results by paving the way for *de novo* addition of gene within the precise functional boundaries of gene promoters and other operational elements of genetic segment. The scientific community discusses different scenarios arising by incorporating the utilization of CRISPR/Cas9 enzymatic system (Cas9 is a kind of caspase) or using the jumping genes action (whereby genes switch locations within a genome; may be inverted segmentation) that may prove devastating in terms of unknown and unexpected gene transfer. However, research has shown very few negligible chances of such a genetic recombination event (due to the presence of spacer in-between *de novo* segments) but cannot be ignored. The benefits of CRISPR/Cas9 are diverse such as short breeding cycle attainment within the same range of atmospheric conditions and higher yield (Ma et al., 2014).

### 4.3 The potential of next-generation sequencing

The dynamism of agriculture sector is relied also on the crop improvement programs. These include breeding better crops, bio-fortifying crops, understanding the complete genetic make-up, and functional regulation of genes involved. Of all these, it is the functional regulation of genes whose deeper understanding is required in order to fill in the gaps between different sectors of system biology of the concerned agriculturally economic important product.

In case of the role of next-generation sequencing technologies, the inputs provide ascertain fresh gene annotations, thereby focusing the paradigm of information toward the metabolite; so the production may reveal the crucial points of biochemical interactions. Similarly, nickel (Ni^2+^) is the element that binds phytate molecules in crystalloid bodies of mustard seed and enhances the action of phytic acid in lowering the bioavailability of proteins present when included in human diet. Moreover, a deeper understanding of phytic acid genetic elements becomes crucial and, for that gene sequence, must be validated out. That validation is supported by next generation sequencing methodologies, whose tissue-specific gene expression is highly favored in that area of seed. Moreover, the regulation of other enzymes concerned with Ni^2+^ (divalent in this case) can be linked to switching ON/OFF expression of those genes that provide enzymatic machinery for phytic acid biosynthetic pathway. This establishes a picture of *in vivo* functioning molecular machinery in *in silico* mode. However, to reach that point, one has to start with next-generation sequencing technologies, whose revilement of genetic sequences makes other bioinformatic steps possible (Liu et al., 2023).

The word “next-generation sequencing” depicts the sequencing techniques apart only from the first generation sequencing methodology (Qin, 2019).

## 5 Revealing the pioneering molecular mechanisms for the production of biostimulants by sequencing strategies

The technique of isotopic labeling provides much tricky shortcut revealing about the presence of biochemicals, especially secondary metabolites as major biostimulants with the first one drafted in late 1950 (Bentley, 1999). However, how gene products are synthesized within the cellular locations is a mystery until researchers utilize gene expression models. Thereby, it becomes an important milestone to cover for, and the story began with landmark groundbreaking articles gifted to the scientific world by logistic scientific minds from 1984 to 1991 (Malpartida and Hopwood, 1984; Cortes et al., 1990; Donadio et al., 1991). By this time, it became clear with the advent of rise in molecular biology gene annotation tools viz., nucleotide cloning and sequencing strategies, which were searched in stretch of DNA obtained from Actinomycetes bacterial species that are responsible for the production of secondary metabolites serving as biostimulants. Actinomycetes are naturally resistant to environmental extreme conditions such as extreme temperature and pH, and it is the production of secondary metabolites that make them tough in response to adverse environmental conditions. This clears the picture of the importance of secondary metabolites. On one hand, secondary metabolites fulfill the role of bacterial survival, and on the other hand, they serve as biostimulants. These molecular revealing are made through high-throughput genome sequencing strategies. Thereby adopting the next-generation sequencing strategies, the genes were found to be organized in gene clusters, the very first one deciphered was that of a fungus, *Penicillium chrysogenum*. The clusters identified were associated with the synthesis of penicillin (a broad range antibiotic, acting as biostimulants by killing heaps of pathogenic bacteria) and an enzyme named L-*δ*-(*α*-aminoadipoyl)-L-cysteinyl-D-valine (responsible for large-scale peptide synthesis by a nano-meter scale fungi cell, acting as biostimulants by sustaining the growth of such fungal species by producing the enzyme) (Díez et al., 1990). Similar molecules serving as biostimulants were found to be mycotoxins (metabolites produced by fungi responsible for eliminating pathogens of plants). Interestingly, these molecules falls under the class of terpenes and terpenoid-related compounds, of which the special mention is trichothecenes (Hohn et al., 1993). Like penicillin acting as a broad-range antibiotic, the aflatoxin, broad-range anti-fungal compound synthesis, is perceived by *Aspergillus* sp. (Yu et al., 2004).

The cosmid libraries were first used before pre-genomic era, where knocked-out mutants were screened in fungi (Mayorga and Timberlake, 1990; Hendrickson et al., 1999). This was followed by an insertional mutagenesis approach using plasmid recovery (Yang et al., 1996; Chung et al., 2003). However, these are the cDNA expression library, reverse transcriptase polymerase chain reaction (RT-PCR), and subtractive hybrid PCR with suppression analysis, which changed the secondary metabolite screening approach upto the gene level and took it to the accurate prediction of the products at the gene cluster level (Beck et al., 1990; Linnemannstöns et al., 2002; O’Callaghan et al., 2003). These represented a trait of the organism marked by a particular gene or gene cluster level. However, what has limited the screening strategy here is the large genome size which requires high throughput methods. Moreover, the partial gene clusters were selected to deal with candidate genes like that of polyketide production, which naturally influenced the rhizosphere to be a biostimulant in root zone. The development of the PCR methods such as degenerated primers at both flanking ends of DNA revealed the conserved ketosynthase enzymatic domain with many functions to play for with a slight change in the secondary structure of protein domains and motifs and represented by a single nucleotide polymorphism. This opened the gateways for further cosmid library screening, and finally, large dataset gene clusters were screened to a single change in nucleotide stretch (Chooi and Tang, 2012). These methods revealed the production of fumotoxins (a type of mycotoxin inhibiting pathogenic bacteria growth) and aflatoxins with various classes and sub-classes, thereby making it easy to understand the functional dynamism of gene clusters. To contribute to phylogenomic analysis, the pioneering steps were taken, which resulted in polyketide gene synthesis with eight genome lineages widely spread in fungal kingdom. The use of degenerated primers and cosmid library screening was helpful as they identified partial gene clusters. All these steps were taken prior to whole genome sequencing, as the overlapping fragments may represent the false-positive target hits. Some examples of the fungal biostimulants expressed by candidate genes that were screened are tromin, tryptoquivaline, echinocandinin, and prenyl atedxanthones, which were obtained from *Dothistroma septosporum*, *A. clavatus*, *Emericella rugulosa*, and *A. nidulans*, respectively (Gao et al., 2011; Cacho et al., 2012). To add to the complexity of biostimulants, there are some overlapping genes which tend to produce a different product but with efficient biostimulant capability. Some examples are fumagillin, pseurotin, and fumitremorgin (Wiemann et al., 2013). The illustrative pioneering studies were performed in genomes of the genus *Neurospora*, *Botrytis*, and *Cochliobolus* (Kroken et al., 2003).

To add to the knowledge of screenings of biostimulants produced by animals and plants, there is a need to identify secondary metabolites involved and the gene clusters, if present for such isolates and exudates (Vioque et al., 2000). Since the bio-degradation potential and sub-fractionation are more commonly associated with plants and animal-led biostimulants, the approaches must target individual gene expression patterns in relation to real-time PCR analysis. These are some of the reasons why the mechanisms behind biostimulant-led secondary metabolite action are not much known in plants and animals, as they are by-products of secondary metabolite or the bio-degradation byproduct of secondary metabolites, which are not specified due to a diverse array of biochemicals involved. In case of the role of next-generation sequencing technologies, the connecting links could be established between gene, gene product, by-product enhanced in nature, and, finally, the biostimulant action.

## 6 Challenges, future prospects, and conclusion

By focusing on the atomic systems that underpin the observed exercises, ranchers will also find it easier to separate the physiological and plant digesting routes involved in this cycle. In addition to being a promising practice that is good for the environment, the use of biostimulants may decrease the usage of agrochemicals (such as mineral fertilizers and chemicals for controlling pests and pathogens) and raise the efficiency with which natural resources are utilized. Additionally, it has the potential to increase the amount of food that is available to feed the world’s expanding population and the sustainability of agricultural and horticultural production systems. The most recent information on the categorization of bio-stimulatory agents, their main mechanism of action, and their practical uses in agricultural productivity are presented in this article. Despite the fact that there are many situations in which the administration of a biostimulant benefits the plant and production, more research is needed for the application techniques because it seems necessary to address product and crop specificities.

The variable effects that have been described in the literature are a result of uncertainties in application times, procedures, and doses for biostimulants, which are natural matrices that contain a variety of chemicals from different classes and activities. Last but not least, the crop factor is also important because the genotype greatly influences how people react to biostimulant products, particularly in situations when reactions are unknown and the conditions necessary for the same are present. Taking into consideration the past, new biostimulant products are necessary since the creation and use of biostimulant is a developing relationship. However, this should be addressed utilizing a different approach that emphasizes the synergistic benefits of many biostimulatory specialists rather than administering a single item. By focusing on the atomic systems that underpin the observed exercises, ranchers will also find it easier to separate the physiological and plant digesting routes involved in this cycle. In addition to being a promising practice that is good for the environment, the use of biostimulants may decrease the usage of agrochemicals (such as mineral fertilizers and chemicals for controlling pests and pathogens) and raise the efficiency with which natural resources are utilized. Additionally, it has the potential to increase the amount of food that is available to feed the world’s expanding population and the sustainability of agricultural and horticultural production systems.

The various secondary metabolites secreted by plants are of immense importance to humans as they are utilized as drugs against potent diseases, eradicate pathogenic microorganism from the soil which prevents the stimulatory growth of plant, may be used to cure viral infections within the plant-soil system, and regulate the moisture levels in maintaining the humidity. Apart from the above mentioned biological significance imparted by PB, their importance extends to the level of pest control as insecticidal, nematicidal, and fungicidal agents present in nature, which is regulated by nature and produced by plants.

Innovative technologies are continued to work on showing the entire genomic sequence in order to expand the screening of PBs. The availability of the metabolite is ultimately determined by quantification once it has been genetically ruled out and its expression yield has been determined molecularly. The possibilities for investigating PBs are virtually limitless, starting with chromatographic techniques such as Ultra High Performance Liquid Chromatography (UHPLC) by Corporation and continuing through the analysis between nuclear magnetic resonance (NMR) and X-ray crystallography.

## Author contributions

SG: Writing – original draft, Writing – review & editing. PN: Writing – original draft, Writing – review & editing. AK: Conceptualization, Methodology, Writing – review & editing. SJ: Conceptualization, Formal analysis, Investigation, Supervision, Writing – review & editing. HP: Conceptualization, Investigation, Supervision, Writing – review & editing. PS: Conceptualization, Formal analysis, Writing – review & editing. SS: Conceptualization, Funding acquisition, Writing – review & editing. MA: Funding acquisition, Supervision, Writing – review & editing. UA: Conceptualization, Funding acquisition, Writing – review & editing. AM: Conceptualization, Formal analysis, Writing – review & editing.

## Footnotes

^1^http://www.illumina.com/systems/sequencing.html
